# Special Issue “Construction Materials and Other Related Materials: Basic Theory, Applied Technology and Advanced Research Methods”

**DOI:** 10.3390/ma17010057

**Published:** 2023-12-22

**Authors:** Xi Du, Youliang Chen, Rafig Azzam, Tomas Manuel Fernandez-Steeger, Bin Peng

**Affiliations:** 1 Department of Civil Engineering, University of Shanghai for Science and Technology, Shanghai 200093, China; pengtj77@hotmail.com; 2 Rheinisch-Westfälische Technische Hochschule Aachen, 52056 Aachen, Germany; azzam@lih.rwth-aachen.de; 3 Institut für Angewandte Geowissenschaften, Technische Universität Berlin, 10587 Berlin, Germany; fernandez-steeger@tu-berlin.de

## 1. Introduction

The rapid advancement of science and technology has facilitated the creation of new technologies and techniques, leading to the convergence and diversification of fields. Consequently, these innovations have experienced growing use in the production of construction materials, resulting in an ongoing and dynamic revolution within the industry. The ongoing research in construction materials consistently strives to be at the forefront of technological advancements. Drawing upon the fundamental principles of mechanics, this approach continually incorporates the most recent research discoveries from mechanics and related fields, enhancing the disciplinary knowledge reservoir and adeptly tackling engineering challenges. This Issue encompasses theoretical and applied research contributions, with a particular emphasis on fostering collaboration across other disciplines [1,2,3,4,5,6,7,8,9,10,11,12,13,14,15].

Investigating mechanical properties in construction materials has undergone substantial interdisciplinary development alongside contemporary engineering science and technology. This field has emerged as a fundamental basis for progress in various domains, including aviation and aerospace, machinery, automation technology, materials and processing, and biomedical material science. The mechanical characteristics of construction materials demonstrate a broad spectrum of features, which are both intricate and diverse. These attributes result from the multidisciplinary nature of their creation and the reciprocal influence they exert across different sectors. The resolution of significant engineering and technological challenges is achieved using the vital and indispensable role played by mechanics [16,17,18].

A classification scheme was created based on the themes and areas of attention of the study publications. The 15 papers in this Special Issue were divided into four general categories:

(1) Multi-Phase Materials and Enhancers, which investigated the analysis and evaluation of mechanical properties and performance enhancement methods for various materials [2,3,5,9];
(2) Geotechnical Mechanics and Soil Engineering, which examined topics like rock mechanics, swelling pressure–suction relationship, grout properties, and excavation-induced ground response [1,6,7,14];
(3) Concrete Mechanics and Materials, which concentrated on the effects of organic matter components, constitutive relationships, and concrete strength [4,8,15];
(4) (Hydro-mechanics, Groundwater, and Slope Stability, involving the study of the time-dependent effect of seepage force on hydraulic fracture initiation and the failure mechanism of slopes during heavy rainfall events [11,12,13].

This classification offers a thorough overview of the many topics covered in material engineering, including slope stability, hydro-mechanics, geotechnical mechanics, performance enhancement, and multi-phase materials.

## 2. Short Description of the Articles Presented in This Special Issue

The topics of the original research papers published in this Special Issue of Materials are derived from almost 30 international universities and scientific centers.

Chen et al. [1] developed a framework for assessing the chemical damage caused by water–rock interactions, drawing upon the principles of chemical kinetic theory. The authors incorporated the concept of damage mechanics theory to define the chemical damage factor, considering the pH and duration of the interaction. However, they did not account for the temporal aspect of the damage. Additionally, they quantified the water damage variable by measuring the quantity of soluble cement lost due to the rock–water reaction. Furthermore, they introduced a force damage variable by employing the Weibull distribution. The SMP criterion was used as a measure of rock strength, incorporating the influence of water chemistry and peritectic pressure. To construct the rock chemical corrosion–water–peritectic pressure damage ontology model, Lemaitre’s assumption of strain equivalence was considered. The parameters of the model were determined through theoretical derivation. The comparison between the theoretical and experimental curves derived from the developed damage constitutive model demonstrates a strong agreement. Consequently, this model offers valuable theoretical insights for designing, operating, constructing, and maintaining real-world rock projects exposed to water-chemical corrosion.

According to the research conducted by Chen et al. [2], the authors observed that incorporating red mud-based stabilized soil, along with nano-SiO_2_ and other additives, leads to improved strength characteristics, suitable for roadbed construction. Incorporating nano-SiO_2_ into gypsum and cement demonstrates a synergistic phenomenon, leading to a notable enhancement in the initial unconfined compressive strength. The observed increase in strength exceeds the established criteria for roadbase materials. The specimens exhibit a compressive deformation behavior that showcases a blend of elastic and plastic characteristics, with the presence of both brittle damage and ductile behavior. The utilization of red mud as a predominant constituent in stabilized soil offers a feasible and ecologically sustainable strategy for managing industrial waste, exhibiting considerable promise for its application in the filling of roadbeds. These insights significantly contribute to the field of material engineering and establish the groundwork for progress in the construction of sustainable infrastructure.

The research conducted by Chen et al. [3] emphasizes the efficacy of synergistically enhancing red mud-based stabilized soil by incorporating nano-SiO_2_ and gypsum. Subjecting the soil to a 7-day curing process demonstrates satisfactory compressive strength characteristics, fulfilling the prescribed standards for road subgrade materials. This desirable outcome is achieved by including nano-SiO_2_ at a concentration of 1%, which represents the most advantageous combination. The changed material exhibits greater strength, as seen by the enhanced structural compactness and increased presence of needle-like and columnar calcium alumina, which can be observed via microscopic analysis. The curing process encompasses hydration and pozzolanic reactions, assisted by the promotion of nano-SiO_2_ and the enhancement of gypsum. This paper presents a novel model for the initial stages of hydration, showcasing the advantageous effects of nano-SiO_2_ and gypsum alteration. This study offers significant contributions to the understanding and implementation of sustainable practices in the exploitation of red mud, with a specific focus on reducing its environmental consequences. Additionally, it presents potential opportunities to apply red mud in road construction.

The present study conducted by Shao et al. [4] examines the impact of organic matter constituents, namely fulvic acid (FA) and humic acid (HA), on the mechanical characteristics of cemented soil. The research findings indicate the association between an increase in the FA/HA ratio with a decrease in the uniaxial compressive strength of cemented soil while concurrently enhancing its compressibility. The prolongation of the curing duration resulted in the augmentation of the cement hydration process, leading to an elevation in strength. The strength and deformation properties were more significantly influenced by fulvic acid than humic acid. The inclusion of organic matter impeded the process of cement hydration and caused disturbances in the soil structure, hence impacting its mechanical qualities related to strength and deformation. The initial resistivity and structural property parameters decreased with increasing FA/HA and increased with age. The study successfully established quantitative correlations among strength, deformation characteristics, the ratio of fine aggregate to hydraulic aggregate (FA/HA), and the duration of curing. In general, this study offers significant insights into the selection of optimal curing time and organic matter levels in practical applications requiring cemented soil.

The study conducted by Wang et al. [5] revealed that the application of polyacrylic (PA) effectively acts as a passivator for water-sensitive expanding soil (ES). The research findings indicate that the incorporation of PA into ES composites can effectively mitigate water sensitivity, resulting in reduced swelling and shrinkage deformation, as well as improve composite strength. Composites containing 6% PA content exhibit optimal suppression of deformation and achieve saturated shear strength. The physical, mechanical, and deformation properties of PA-ES composites are enhanced through the process of PA passivation, achieved through adsorption, binding, and filling. The addition of PA alters the distribution of particle sizes, resulting in a decrease in the reactivity of clay particles and the optimization of the composite gradation. Phosphoric acid exhibits potential as a soil passivator for expansive soils, providing an engineering point of reference in order to address the issues associated with these types of soils. The water-sensitive passivation effect of PA-ES composites contributes to developing sustainable and resilient infrastructure projects by effectively mitigating water sensitivity problems associated with ES.

Wang et al. [6] studied compacted bentonite’s swelling pressure under conditions of hydration. Research results demonstrate that swelling pressure increases linearly with dry density. Dry density affected swelling pressure–suction curve heterogeneity. Lower-density specimens showed this tendency to a far greater extent. Swelling pressure–suction curves were divided into unsaturated and saturated regions during the analysis. Attention was drawn to a critical saturated state (CSS) curve that remained invariant under stress. The paper also presented a novel method for constructing bentonite-specific constitutive models. 

The impact of grout annulus (GA) [7] qualities on the tensile performance of rockbolts is emphasized by Chen et al.’s research. Their paper assesses the impact of GA on rockbolt behavior via numerical simulations and in situ roadway reinforcement examples using modified cable elements. The findings show that higher shear strength (SS) of the GA is followed by increased displacement at the peak force, increasing the peak force in rockbolts. Accurate modeling of this behavior is made possible by the improved cable elements’ adoption of a two-stage coupling law. The peak force in rockbolts is substantially influenced by the SS of the GA, with larger SS values resulting in higher forces. 

The present work by Wu et al. [8] elucidates the findings derived from an investigation into the effects of high-temperature treatment of ordinary concrete as well as concrete treatment with nano-titanium dioxide. Both types of concrete exhibited a propensity to undergo splitting failure after exposure high temperatures. The rise in temperature led to a decline in the mechanical characteristics of the concrete, particularly in terms of compressive resistance and secant modulus. The incorporation of nano-titanium dioxide resulted in an enhancement of the thermal stability of the concrete when subjected to temperatures lower than 600 °C. Nevertheless, there was no significant discrepancy observed in the mechanical properties of the two concrete types when exposed to temperatures over 600 °C. The stress–strain curves of the specimens exhibited similar forms and characteristics both before and following exposure to elevated temperatures. The aforementioned observation played a crucial role in the formulation of a constitutive relationship that incorporated the factor of high-temperature damage. The current investigation provides valuable insights into the behavior and performance of concrete structures when exposed to high temperatures. 

Yoon et al. [9] investigate the effects of synthesis of polyurethane on glass beads (GB) in terms of improve functionality and assess the mechanical properties of composites with polymerized particles (PUGB) in this study. The results demonstrate that polymer coating increases the contact area and maximal void ratio, resulting in a smoother surface. With an increase in polymerized particle content (*C*_PU_), the constrained modulus (*M*) decreases, while the shear wave velocity (*V*s) decreases due to a reduction in contact stiffness. Comparing PUGB-GB and rubber–sand mixtures, PUGB exhibits a lesser decrease in *M* while retaining a comparable shear modulus. The study emphasizes the potential application of polymer coating in improving the performance of PUGB-GB mixtures and suggests further investigation of their dynamic behavior in a variety of applications.

The investigation conducted by Xu et al. [10] aimed to examine the utilization of steel slag powder (SSP) to augment the heat release and self-healing characteristics of asphalt and asphalt mixes. The results indicated that the microwave heating capabilities of SSP were enhanced as a result of its metal oxide composition. The appropriate healing temperature was calculated, and the asphalt mixtures using SSP replacements exhibited enhanced healing indexes. The researchers determined that temperature emerged as the primary influential component in the study. Furthermore, it was seen that the total healing index declined as the duration of damage grew. Nevertheless, the SSP mixture consistently maintained a healing index exceeding 50% even after undergoing multiple mechanical damage. The present study emphasizes the potential of SSP in improving the properties of asphalt, thus offering valuable insights for road maintenance and prolonging the lifespan of pavements.

The present study conducted by Rim et al. [11] presents a novel seepage model and circumferential stress calculation model in order to examine the time-dependent impact of seepage force on the beginning of fractures. The results indicate that the suggested models effectively forecast the pressure at which fractures begin while also highlighting the contrasting impacts of poroelastic stress and seepage-induced tensile stress. Over time, the overall circumferential effective stress diminishes, transitioning into tensile stress and thereby augmenting the probability of fracture initiation. The initiation of fractures is temporally controlled by two key factors: hydraulic conductivity and fluid viscosity. The findings presented in this study provide contributions to our understanding of the dynamics of fracture initiation caused by seepage.

Zhang et al. [12] employ physical model experiments to investigate the Pengshan Landslide occurring within a basalt platform region. The objective is to ascertain the manner in which slopes undergo morphological alterations and experience failure under conditions of intense precipitation. During the testing process, real-time monitoring is conducted to assess pore water pressure, earth pressure, and slope deformation. The stability of a slope is determined via empirical investigations that examine the impact of intense precipitation events and the presence of a vulnerable interlayer on slope behavior. The empirical evidence demonstrates that the frequency of slope instability occurrences in basalt platforms is significantly influenced by two key factors: the rainfall intensity and the existence of weak interlayers. The outcomes of this study could potentially enhance our comprehension of temporal variations in slope dynamics within regions characterized by basaltic platforms.

Zhang et al. [13] investigate the shear creep properties of rock gaps with various 3D configurations. The results provide crucial insight into how these morphological characteristics influence the behavior of shear creep. According to the results, as the 3D morphological measure increases, failure lasts for a shorter duration and damage increases. Additionally, as the 3D morphological parameter (represented by *Z*_2_) increases, creep deformation and creep rate decrease. The research also demonstrates that there is a linear relationship between the long-term strength of rock discontinuities and the 3D morphological parameter. In order to more accurately characterize the observed behavior, the study suggests a model that incorporates a damage variable and is based on the nonlinear Burgers model. This model effectively illustrates how discontinuities increase creep and correlate with experimental data at various stress levels.

The present study conducted by Wang et al. [14] examines the impact of volumetric block proportion (VBP) on the ground reaction generated by excavation in rock masses resembling talus formations. This investigation employs two-dimensional discrete element method (DEM) simulations. Through the examination of micro and macro attributes, this research discerns three unique categories of talus-like rock formations, distinguished by varying values of VBPs. The research emphasizes the significance of employing rigid lining support systems to ensure the preservation of ground stability in soil-type and hybrid-type talus-like rock formations. The findings above offer valuable insights into the optimization of support schemes during tunnel excavations in diverse talus-like rock masses, with the ultimate goal of ensuring the safety and efficiency of construction activities.

The study conducted by Zhang et al. [15] offers contributions to understanding the various elements that impact the mechanical properties of alkali-activated slag materials. The findings of the study demonstrate that the proportion of the alkali activator is a crucial factor in determining the ultimate level of strength. Furthermore, it can be shown that the solid-to-liquid ratio and water–cement ratio exhibit negligible influence on the rate of strength development. Nevertheless, an elevated modulus of the alkali activator, coupled with a water–cement ratio of around 0.4, results in enhanced strength. Moreover, the augmentation of the aluminum content by means of metakaolin substitution results in an improvement in the development of late-stage strength and toughness. The aforementioned findings provide valuable insights on customizing alkali-activated slag materials to suit certain applications. Engineers possess the ability to utilize these observations to enhance the material qualities through the selection of an acceptable mix ratio and the adjustment of the composition of the slag components. In general, this study enhances the ability to tailor and adapt alkali-activated slag materials for a wide range of technical uses.

## 3. Conclusions

The decision to allocate a Special Issue to the subject matter of “Construction Materials and Other Related Materials: Basic Theory, Applied Technology, and Advanced Research Methods” was prompted by the wide range of existing research on this area. This Special Issue provides an in-depth examination of the mechanical properties exhibited by construction materials, which are distinguished by a multitude of intricate and varied characteristics. The aforementioned features arise from the multidisciplinary nature of the processes implicated in their formation and the interplay among different sectors. The Issue also highlights the significance of mechanics in addressing engineering and technical problems as a crucial factor in effectively tackling significant challenges.
